# FgVps9, a Rab5 GEF, Is Critical for DON Biosynthesis and Pathogenicity in *Fusarium graminearum*

**DOI:** 10.3389/fmicb.2020.01714

**Published:** 2020-08-04

**Authors:** Chengdong Yang, Jingjing Li, Xin Chen, Xingzhi Zhang, Danhua Liao, Yingzi Yun, Wenhui Zheng, Yakubu Saddeeq Abubakar, Guangpu Li, Zonghua Wang, Jie Zhou

**Affiliations:** ^1^Fujian Province Key Laboratory of Pathogenic Fungi and Mycotoxins and College of Life Sciences, Fujian Agriculture and Forestry University, Fuzhou, China; ^2^State Key Laboratory of Ecological Pest Control for Fujian and Taiwan Crops, Fujian Agriculture and Forestry University, Fuzhou, China; ^3^Department of Biochemistry, Ahmadu Bello University, Zaria, Nigeria; ^4^Department of Biochemistry and Molecular Biology, University of Oklahoma Health Sciences Center, Oklahoma City, OK, United States; ^5^Institute of Oceanography, Minjiang University, Fuzhou, China

**Keywords:** FgVps9, guanine nucleotide exchange factor, endocytosis, pathogenicity, DON, *Fusarium graminearum*

## Abstract

Rab GTPases play an important role in vesicle-mediated membrane trafficking in eukaryotes. Previous studies have demonstrated that deletion of *RAB5*/*VPS21* reduces endocytosis and virulence of fungal phytopathogens in their host plants. However, Rab5 GTPase cycle regulators have not been characterized in *Fusarium graminearum*, the causal agent of *Fusarium* head blight (FHB) or head scab disease in cereal crops. In this study, we have identified and characterized a Rab5 guanine nucleotide exchange factor (GEF), the Vps9 homolog FgVps9, in *F. graminearum*. Yeast two hybrid (Y2H) assays have shown that FgVps9 specifically interacts with the guanosine diphosphate (GDP)-bound (inactive) forms of FgRab51 and FgRab52, the Rab5 isoforms in *F. graminearum*. Deletion of *FgVPS9* shows impaired fungal growth and conidiation. Pathogenicity assays indicate that deletion of *FgVPS9* can significantly decrease the virulence of *F. graminearum* in wheat. Cytological analyses have indicated that FgVps9 colocalizes with FgRab51 and FgRab52 on early endosomes and regulates endocytosis and autophagy processes. Gene expression and cytological examination have shown that FgVps9 and FgRab51 or FgRab52 function in concert to control deoxynivalenol (DON) biosynthesis by regulating the expression of trichothecene biosynthesis-related genes and toxisome biogenesis. Taken together, FgVps9 functions as a GEF for FgRab51 and FgRab52 to regulate endocytosis, which, as a basic cellular function, has significant impact on the vegetative growth, asexual development, autophagy, DON production, and plant infection in *F. graminearum*.

## Introduction

Rab proteins are small (21–25 kDa) monomeric GTPases/guanosine triphosphate (GTP)-binding proteins and constitute the largest subfamily of Ras-like GTPases (Mizuno-Yamasaki et al., 2012; Li and Marlin, 2015; Pfeffer, 2017). Endocytosis is a temperature-, time-, and energy-dependent process by which eukaryotic cells internalize extracellular fluids, other substances, as well as transmembrane proteins into cytoplasmic vesicles (Munn, 2000; Sorkin and von Zastrow, 2009; Goode et al., 2015), and endocytosis plays indispensable roles in cell polarity, signal transduction and plant infection in phytopathogenic fungi (Fuchs et al., 2006; Higuchi et al., 2009; Ramanujam et al., 2013; Qi et al., 2016; Li L. et al., 2017). Rab5 is the best documented Rab protein involved in the early endocytic pathway, which promotes the fusion of early endosomes by interacting with its effectors (Ohya et al., 2009). Rabs exert their functions by alternating between active GTP-bound and inactive guanosine diphosphate (GDP)-bound states, and this cycle is regulated by cognate guanine nucleotide exchange factors (GEFs) and GTPase-activating proteins (GAPs) (Barr and Lambright, 2010). GEFs facilitate GDP release and GTP binding for Rab activation, which in turn interacts with downstream effectors to promote multiple functions in vesicular transport, including vesicle formation, movement on cytoskeleton, membrane tethering, and fusion. In contrast, GAPs accelerate the intrinsic Rab GTPase activity, converting GTP-bound active state to its GDP-bound inactivate state (Mizuno-Yamasaki et al., 2012; Li and Marlin, 2015; Pfeffer, 2017).

Three types of conserved Rab GEF domains (VPS9, SEC2, and DENN domains) have been reported. There are more than 40 Rab GEFs in humans; most of them possess at least one of these GEF domains (Ishida et al., 2016). Vps9, a class D Vps protein in yeast, is involved in vesicle-mediated vacuolar protein sorting and shows GEF activity toward Vps21/Rab5 (Burd et al., 1996; Esters et al., 2001). Three Vps9 domain-containing proteins (Vps9, Muk1, and Vrl1) have been identified in the budding yeast *Saccharomyces cerevisiae*; they have distinct but overlapping functions. Both Vps9 and Muk1 act as GEF for Vps21, Ypt52, and Ypt53 and participate in the vegetative growth, stress response, vacuole formation, and Golgi-endosome trafficking pathway. Moreover, Vps9 and Muk1 predominantly localize to the cytosol in wild-type cells (Hama et al., 1999; Paulsel et al., 2013; Bean et al., 2015). Three Vps9 domain-containing proteins (PoVps9, PoMuk1, and PoVrl1) have been identified in *Pyricularia oryzae*, and both PoVps9 and PoMuk1 promote the crosstalk between endocytosis and autophagy processes through activation of PoVps21. Moreover, they have overlapping functions in the vegetative growth, conidiation, and pathogenicity in host plants (Zhu et al., 2018). FolVps9 also shows GEF activity on FolVps21 in *Fusarium oxysporum* f.sp. *lycopersici* (*Fol*) where deletion of *FolVPS9* phenocopied the single- and double-deletion mutants lacking Vps9 homologs in *P. oryzae* (Li et al., 2019).

*Fusarium graminearum* (teleomorph: *Gibberella zeae*) is the causal agent of *Fusarium* head blight (FHB) or head scab disease on a variety of cereal crops (McMullen et al., 1997; Goswami and Kistler, 2004). This disease does not only result in yield and quality losses but also produces trichothecene mycotoxins such as deoxynivalenol (DON), zearalenone, and nivalenol in infected grains, among which DON is most prevalent, thus imposes serious threats on human and livestock health (Kurata and Ueno, 1984; Pestka and Smolinski, 2005). DON production is sequentially controlled by a series of *TRI* genes, and these genes exist in clusters in *F. graminearum* and *Fusarium sporotrichioides* (Brown et al., 2004; Alexander et al., 2009; Boenisch et al., 2017). Tri6 and Tri10, two major transcriptional regulators, regulate the expression of almost all *TRI* genes. In addition, these two proteins also regulate DON production via the cyclic adenosine monophosphate (cAMP) signaling pathway (Seong et al., 2009; Jiang et al., 2016). DON is also a virulence factor in *F. graminearum*. Deletion of *FgTRI5* abrogates DON production and significantly reduces the fungal virulence on wheat (Proctor et al., 1995; Desjardins, 1996). In recent years, it has become clear that Rabs and their regulators and effectors play a critical role in DON production and pathogenicity in *F. graminearum*. DON production and infection in plants decrease significantly in the knockout mutants of *FgRAB8* and its GEF (*FgSEC2A*), *FgRAB7* and its GEF (*FgMON1*), and the effector (*FgVPS41*). Furthermore, deletion of endocytosis-related genes *FgRAB51* and *FgRAB52* also blocks the DON production and pathogenicity of *F. graminearum* (Li et al., 2015, 2018; Zheng et al., 2015, 2018a). Nevertheless, the functions of FgRab5 regulators in endocytosis, DON biosynthesis, and virulence on wheat are unclear in *F. graminearum*. In this study, we have identified the FgRab5 GEF FgVps9 and demonstrated that FgVps9 localizes to FgRab5-labeled early endosomes and interacts with FgRab51DN (DN, dominant negative) (FgRab51^N126I^) and FgRab52DN (FgRab52^N133I^). Deletion of *FgVPS9* resulted in impaired vegetative growth, conidiation, and autophagy, as well as defects in endocytosis, DON biosynthesis, and plant infection in *F. graminearum*.

## Materials and Methods

### Strains, Media, and Incubation Condition

All strains (the wild-type PH-1 and all derived mutants strains) are cultured in starch yeast media (SYM) [1% starch (*w*/*v*), 0.6% yeast extract (*w*/*v*), 0.3% sucrose (*w*/*v*), and 2% agar (*w*/*v*)] at 28°C under dark condition. Vegetative growth assays were conducted on solid complete media (CM) [0.6% yeast extract (*w*/*v*), 0.6% casein hydrolyzate (*w*/*v*), 1% sucrose (*w*/*v*), and 2% agar (*w*/*v*)] at 28°C in an incubator for 3 days. For conidial production, the various strains were cultivated in liquid carboxymethylcellulose (CMC) medium, and conidiation was counted as previously reported (Zheng et al., 2012). For conidial germination assay, macroconidia from the tested strains were cultured in liquid CM for 4 h with gentle agitation (Seong et al., 2008). Conidia from the wild-type strain PH-1 and all the mutants were visualized using an Olympus BX51 microscope. For perithecia formation, mycelia plugs of the tested strains were cultured on carrot agar plates at 28°C for 7 days and then rubbed with a sterilized spreader after applying 2.5% sterilized Tween-60 solution to induce sexual reproduction (Leslie and Summerell, 2006). Mycelia cultivated in liquid CM and trichothecene biosynthesis inducing (TBI) media at 28°C were used for DNA and RNA extractions, respectively. For toxisome generation, mycelia were cultured in liquid TBI media as previously described (Boenisch et al., 2017).

### Gene and Domain Deletion, Complementation, and Point Mutation

Analysis of the conserved domains was conducted using the SMART program^1^. Phylogenetic tree construction was performed using MEGA5.2 with amino acid sequences of Vps9 homologs in three different species. The *F. graminearum* protoplast preparation and fungal transformation were based on a previous description (Hou et al., 2002). Deletion of the *FgVPS9* gene was generated by split-marker approach (Catlett et al., 2003). The flanking sequences of *FgVPS9* gene and hygromycin phosphotransferase (Hph) cassette were amplified by their corresponding primer pairs (Supplementary Table S1). The PCR products were then transformed into protoplasts of the wild-type strain PH-1 as reported (Proctor et al., 1995; Hou et al., 2002). Subsequently, the hygromycin-resistant transformants were identified by PCR using primer pairs OF/OR and UA/H853 (Supplementary Table S1) and further confirmed by Southern blot using the Digoxigenin High Prime DNA Labeling and Detection Starter Kit I (Cat. 11745832910, Roche, United States). For complementation assay, FgVps9 native promoter, full CDS, and green fluorescent protein (GFP) fragments were amplified using their respective primer pairs (Supplementary Table S1) and fused together by splicing by overlap extension (SOE)-PCR. The GFP fragment was fused to the N-terminus of FgVps9. Finally, the integrated fragment of *GFP-FgVPS9* was cloned into pKNT plasmid (Zheng et al., 2015) following digestion using *Kpn*I and *Bam*HI restriction endonucleases. The resulting *GFP-FgVPS9* construct was amplified from the plasmid and sent to the company (Sangon, Shanghai, China) for sequencing to verify its successful insertion into the plasmid. The recombinant plasmid was transformed into the Δ*Fgvps9* mutant protoplast. For FgVps9 domain deletion, the native promoter and the flanking sequences of FgVps9 domains were amplified and fused together by SOE-PCR using their respective primer pairs (Supplementary Table S1). The integrated fragment was inserted into a pKNT plasmid and verified by sequencing and then transformed into the protoplast of the Δ*Fgvps9* mutant. For *FgVPS9* point mutation, specific primer pairs (Supplementary Table S1) were designed according to the SOE-PCR. The PCR products were inserted into the pKNT plasmid and verified by sequencing and then introduced into the Δ*Fgvps9* mutant protoplast.

### Quantitative Real-Time PCR

Strains involved in this section were cultivated in liquid TBI and incubated at 28°C under dark condition for 3 days. Total RNA was extracted from mycelia using the RNA extraction kit and further used to performed reverse transcription using a reverse transcription kit (Takara, 6210A) to generate complementary DNA (cDNA). Relative transcription levels were quantified using TB GREEN kit (Takara, DRR820A) using the respective primer pairs (Supplementary Table S1). β-Tubulin gene was used as internal reference gene in this experiment. The data generated were finally calculated using 2^–ΔΔCT^ method as previously reported (Livak and Schmittgen, 2001). Statistical analyses were performed by multiple *t*-tests from three independent repeats using GraphPad Prism at *p* ≤ 0.05.

### Vector Construction

For *FgTRI1-GFP* (FGSG_00071) and *FgTRI4-GFP* (FGSG_ 03535) construct, their native promoter, full CDS and GFP fragments were amplified using their respective primer pairs (Supplementary Table S1) and fused together by SOE-PCR. The GFP fragment was fused to the C-terminus of FgTri1 and FgTri4 like previous description (Menke et al., 2013). Finally, the integrated fragment of *FgTRI1-GFP* and *FgTRI4-GFP* were cloned into pKNT plasmid following digestion using *Kpn*I and *Bam*HI restriction endonucleases. The resulting *FgTRI1-GFP* and *FgTRI4-GFP* constructs were sent to the company (Sangon, Shanghai, China) for sequencing to verify its successful insertion into the plasmid. The recombinant plasmid were transformed into the wild-type and mutant protoplast, respectively.

### Staining and Observation by Microscopy

The various strains were cultured in liquid CM at 28°C for 24 h. For observation of early endosomes, the hyphae from the cultured strains were collected and stained with FM4-64 dye (Cat. T3166, Invitrogen, United States) at the final concentration of 4 μM and incubated for 15 min and finally observed under a laser scanning confocal microscopy. For endocytosis assays, the hyphae of the indicated strains were stained with FM4-64 at the final concentration of 4 μM and visualized under the red excitation (561 nm) by laser scanning confocal microscopy (Nikon, Japan) at different time points. For toxisome formation and visualization in the wild-type PH-1 and the mutants, *FgTRI1-GFP* and *FgTRI4-GFP* constructs, were transformed into each strain. Successful transformants were grown in liquid TBI at 28°C under dark condition for 3 days and then observed under a laser scanning confocal microscopy. To examine toxisome formation *in planta*, conidia suspension from the fluorescent reporter strains were inoculated on wheat coleoptiles, respectively. After incubation at 28°C and keep humidity in chamber for 72 h, epidermis cells from the infected coleoptiles were removed and observed under a fluorescence confocal microscope.

### Pathogenicity and DON Production Assays

Virulence of the indicated strains on wheat coleoptiles were tested as previously described (Jia et al., 2017). Pathogenicity assays on flowering wheat heads were carried out based on a previous description with minor modification (Zhang et al., 2013). Briefly, the mycelia plugs from the wild-type PH-1 and the mutants were inoculated on the flowering wheat heads; then, a plastic bag was used to cap the wheat heads to keep humidity for 1 week. After removing the bags, wheat plants continue to cultivate for another 7 days before examination for typical scab symptoms. To observe the invasive hyphae *in planta*, the mycelia plugs from the experimental strains were inoculated on the lower epidermis of excised wheat leaves, respectively. After incubation at 28°C and 100% relative humidity (RH), the epidermis cells from the infected wheat leaves were observed for invasive hyphae development under fluorescence confocal microscopy at the indicated times. For DON production assay, same number of mycelia plugs from the tested strains were inoculated in liquid TBI media and incubated at 28°C under dark condition for 7 days. DON production was then measured using an ELISA-based DON detection kit (FINDE, Shenzhen, China) (Xie et al., 2019). Briefly, the mycelia were collected, dried, and weighed. The media were used for DON measurement using the detection kit with standard solution. The DON production was standardized as per gram dry weight mycelia.

### Yeast Two Hybrid

For yeast two hybrid (Y2H) assays, the full length cDNAs of Vps21 homologs *FgRAB51/52* (FGSG_05501/FGRAMPH1_ 01G18071, FGSG_11808/FGRAMPH1_01G01731) (Zheng et al., 2015), *FgRAB51/52DN*, and *FgRAB51/52CA* (CA, constitutively active) were amplified by PCR using specific primer pairs (Supplementary Table S1) and the corresponding templates, then inserted into the plasmid pGBKT7 digested with *Nde* I and *Eco*R I to generate bait constructs, respectively. The full-length cDNA of *FgVPS9* (FGRAMPH1_01G01477) was obtained by PCR using the relative primer pairs (Supplementary Table S1) with the wild-type PH-1 cDNA as template. The PCR product was cloned into pGADT7 plasmid digested with *Nde* I and *Eco*R I to create the prey construct. All prey and bait constructs were verified by sequencing (Shanghai, China) and cointroduced into the competent cells of AH109 yeast strain according to a previous protocol (Clontech Laboratories, 2007). Yeast transformants expressing each pair of proteins were assayed for growth on SD/-Leu/-Trp/-His/-Ade and for MEL1 reporter activities. Interaction between pGADT7-T and pGBKT7-53 was used as positive control, and pGADT7-T and pGBKT7-Lam served as negative control.

### Autophagy Assay

To test the autophagy process in the wild-type PH-1 and the Δ*Fgvps9* mutant, *GFP-FgATG8* construct was transformed into the protoplasts of the wild-type PH-1 and the Δ*Fgvps9* mutant, respectively. The GFP-FgATG8 expressing strains were cultured in liquid CM at 28°C for 48 h and then transferred to liquid nitrogen starvation media (MM-N) with 2 mM phenylmethylsulfonyl fluoride (PMSF) for 4 h. Hyphae were stained with 7-amino-4-chloromethylcoumarin (CMAC) and visualized under a fluorescence confocal microscope. For proteolysis assay, mycelia were collected at the indicated time points from which total protein was extracted and Western blot conducted to check the intensity of GFP-FgATG8 protein in the wild-type PH-1 and the Δ*Fgvps9* mutant using anti-GFP antibody (1:5,000, Abcam).

## Results

### Identification of *VPS9* Homolog and Generation of Null Mutant in *F. graminearum*

A *VPS9* homolog was identified in *F. graminearum* by the Basic Local Alignment Search Tool (BLAST) search of the fungal genome database^2^ and was named as *FgVPS9* (FGRAMPH1_01G01477), which contains 2,552 base pairs with four introns and encodes a 783-amino-acid (aa) protein. Domain analysis indicates that FgVps9 contains a Vps9 domain (480–595 aa) and a CUE domain (740–782 aa)^1^ (Supplementary Figure S1A). Phylogenetic analysis of Vps9 proteins in *S. cerevisiae*, *P. oryzae*, *F. graminearum*, and *Fol* indicates that FgVps9 is most closely related to the Vps9 proteins of *Fol* with 86.1% identity, followed by *P. oryzae* with 68.71% identity, and finally *S. cerevisiae* with 40.42% identity (Supplementary Figure S1B), suggesting that the Vps9 proteins may show similar biological functions in filamentous fungi.

To investigate the biological functions of FgVps9 in *F. graminearum*, we created a gene replacement construct using split marker strategy (Supplementary Figure S2A). The mutants were validated by Southern blot analysis, which showed a 4.027-kb band in the *FgVPS9* deletion mutants in contrast to a 5.334-kb band in the wild-type PH-1 (Supplementary Figure S2B). In addition, a *GFP-FgVPS9* expression construct under the control of *FgVPS9* native promoter was generated and transformed into the Δ*Fgvps9* mutant to generate the complementation strain Δ*Fgvps9com*.

### FgVps9 Interacts With the Nucleotide-Free Form of FgRab51 and FgRab52

**FIGURE 1:**
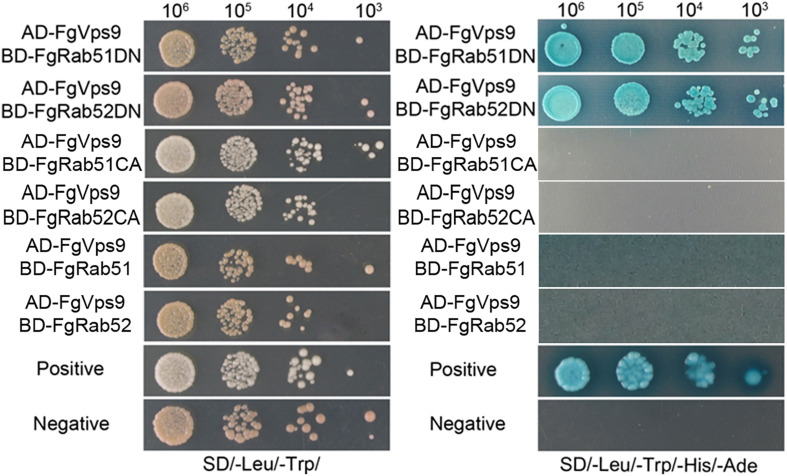
FgVps9 interacts with GDP-bound FgRab51 and FgRab52. Interactions of FgVps9 with FgRab51 and FgRab52, respectively, were examined by yeast two hybrid assays. The interaction between pGADT7-T and pGBKT7-53 was used as a positive control, and pGADT7-T and pGBKT7-Lam served as negative control. Yeast transformants express each pair of proteins were assayed for growth on SD/-Leu/-Trp/-His/-Ade and for MEL1 reporter activities.

Rab GTPases alternate between active GTP-bound and inactivate GDP-bound states. Rab GEFs promote GDP release and GTP binding and usually stabilize the nucleotide-free form with high affinity (Pfeffer, 2001; Grosshans et al., 2006; Hutagalung and Novick, 2011). In *S. cerevisiae*, *P. oryzae*, and *Fol*, Vps9 proteins function as GEFs for the cognate Vps21 (Ypt51) proteins and interact specifically with the nucleotide-free or the GDP-bound form (Hama et al., 1999; Zhu et al., 2018; Li et al., 2019). To determine whether FgVps9 acts as a GEF for FgRab51 and FgRab52 (Vps21 homologs) in *F. graminearum*, we generated a dominant negative (DN) and constitutively active (CA) mutants of *FgRAB51* and *FgRAB52* and determined if they show differential interaction with FgVps9 in yeast two-hybrid assays between AD-FgVps9 and BD-FgRab5DN (nucleotide-free form, FgRab51DN^N126I^ or FgRab52DN^133I^), BD-FgRab5CA (GTP-bound form, FgRab51CA^Q72L^ or FgRab52CA^Q79L^), or the wild-type BD-FgRab51 or FgRab52. Our results showed that FgVps9 interacts specifically with both FgRab51DN and FgRab52DN, but not with the GTP-bound CA mutants or the wild-type FgRab51 and FgRab52 proteins (Figure 1), suggesting that FgVps9 may function as a GEF toward FgRab51 and FgRab52 in *F. graminearum*.

### FgVps9 Plays an Important Role in Vegetative Growth and Asexual Development

**FIGURE 2:**
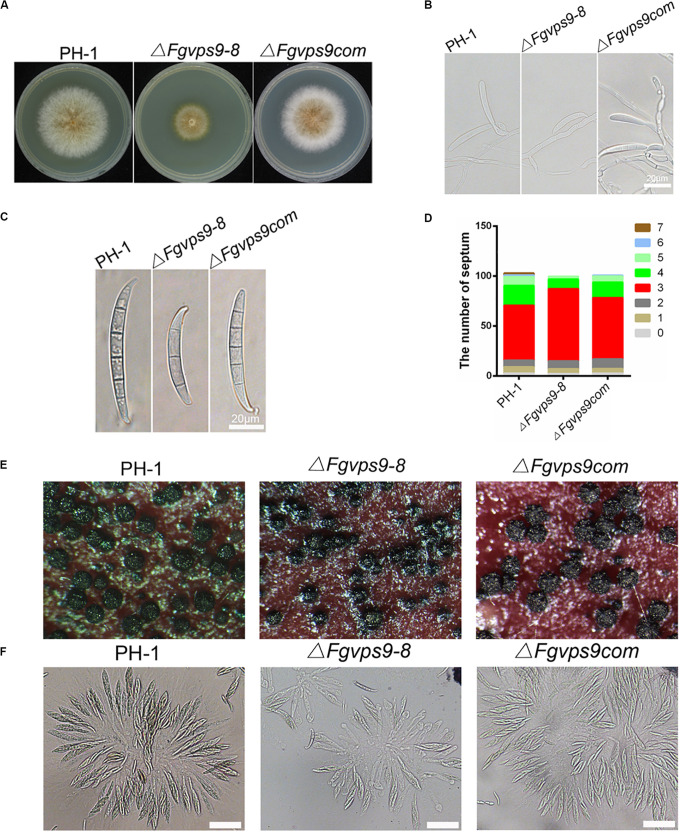
FgVps9 is involved in the vegetative growth and asexual development of *F. graminearum*. **(A)** The wild-type PH-1, Δ*Fgvps9* mutant, and the complementation strain Δ*Fgvps9com* were cultured on complete media (CM) at 28°C for 3 days. **(B)** The wild-type PH-1, Δ*Fgvps9*, and the complementation strain were cultured in liquid CMC media and photographed under a light microscope after 3 days. **(C)** Conidial morphology was observed under a light microscope after the indicated strains were cultured in liquid CMC media for 3 days. **(D)** A bar graph indicating the number of septa in the conidia produced by the indicated strains. **(E)** The perithecia produced by the wild-type PH-1, Δ*Fgvps9*, and the complementation strain after 2 weeks of inoculation on carrot agar plates. **(F)** Images of the ascospores produced by the wild-type PH-1, Δ*Fgvps9*, and the complementation strain taken from a light microscope. Bar = 20 μm.

We next examined the vegetative growth and colony morphology of the Δ*Fgvps9* mutant. After 3 days of growth on CM agar plates, the Δ*Fgvps9* mutant exhibited slower growth and had fewer aerial hyphae compared with PH-1 and the complementation strain (Figure 2A). Furthermore, the colony diameter of Δ*Fgvps9* mutant decreased by 42.6% in comparison to that of PH-1 on CM agar plate. Conidia are believed to play an important role in infecting flowering wheat heads in *F. graminearum* (Stack, 1989; Bai and Shaner, 1994). In order to understand the function of FgVps9 in conidiation, the wild-type PH-1 and Δ*Fgvps9* and Δ*Fgvps9com* strains were cultured in liquid CMC media for 3 days to induce conidia production, followed by microscopic examination. Our results showed that conidiation of the Δ*Fgvps9* mutant was drastically impaired compared to PH-1 and the complementation strains (Figure 2B). Only 1.68 × 10^5^ ml^–1^ conidia were formed by the Δ*Fgvps9* mutant in comparison to 18.34 × 10^5^ ml^–1^ conidia produced by the wild-type strain PH-1. Moreover, 75.4% conidia from the Δ*Fgvps9* mutant were smaller with 3 septa, compared to 56.8% from PH-1 (Figures 2C,D). In addition to conidia, it is shown that the ascospore produced by *F. graminearum* functions in its disease cycle as a primary inoculum (Stack, 1989; Bai and Shaner, 1994). Thus, we examined the sexual development of PH-1, Δ*Fgvps9*, and Δ*Fgvps9com* on carrot agar plates, and found that both perithecia and ascospore from the Δ*Fgvps9* mutant have no significant difference from those from PH-1 and the complementation strains (Figures 2E,F). Collectively, these results suggest that FgVps9 is required for normal vegetative growth and asexual development rather than sexual reproduction in *F. graminearum*.

### FgVps9 Localizes to Endosomes and Is Involved in Endocytosis and Autophagy

**FIGURE 3:**
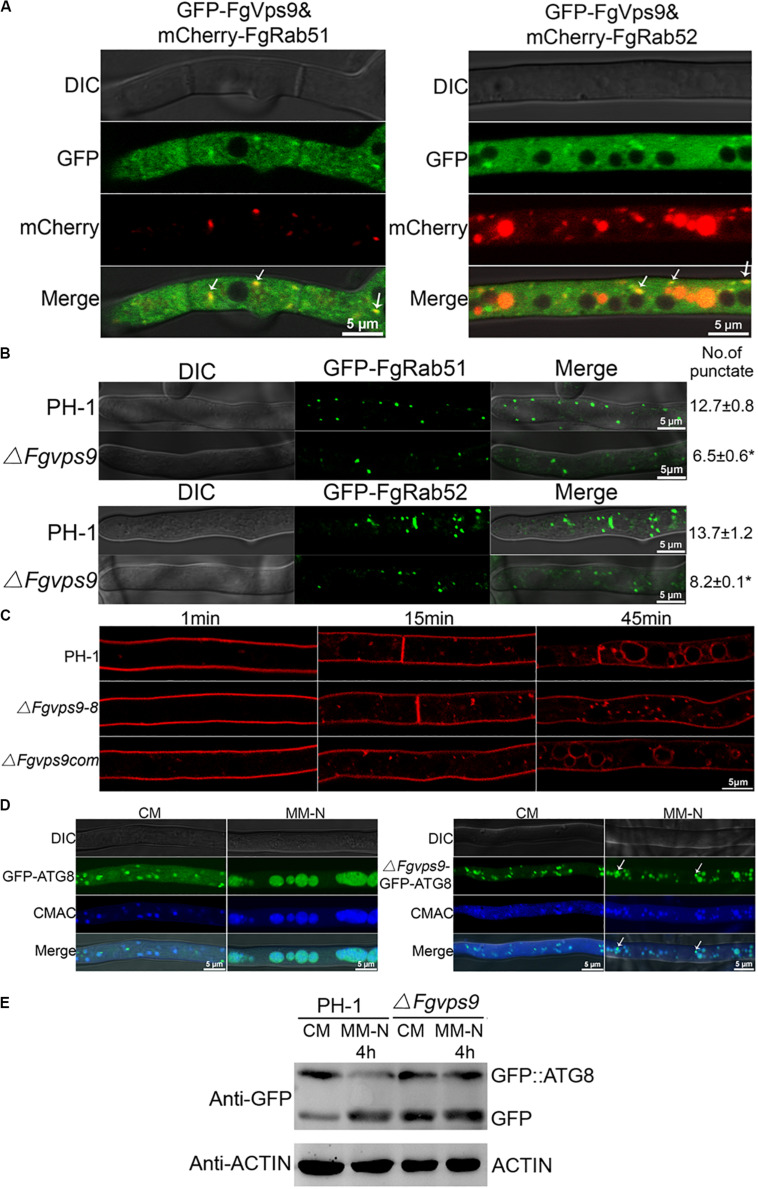
FgVps9 localizes to endosomes and participates in endocytosis and autophagic pathway. **(A)** FgVps9 colocalizes with both FgRab51 and FgRab52 on the endosomes in hyphae. The indicated strains were cultured in liquid complete media (CM) for 24 h. Images were captured from laser scanning confocal microscopy. White arrows indicate overlapping green fluorescent protein (GFP) and mCherry signals. **(B)** The number of FgRab51- and FgRab52-labeled endosomes in the tips of the hyphae in the wild-type PH-1 and Δ*Fgvps9* mutant. The indicated strains were cultivated in liquid CM for 24 h; the number of endosomes at the tips of the 30 hyphae (which were ∼34 μm in diameter each) was counted in each replicate. Statistical differences were calculated by multiple *t*-tests from three independent repeats using GraphPad Prism at *p* ≤ 0.05. **(C)** Hyphae of the wild-type PH-1, Δ*Fgvps9*, and complement strain were incubated in liquid CM for 24 h, then stained with FM4-64 and observed under a fluorescence confocal microscope at different time points. **(D)** Localization of GFP-ATG8 in the wild-type PH-1 and Δ*Fgvps9* mutant, respectively. The indicated strains were cultured in liquid CM at 28°C for 48 h and then transferred to liquid MM-N containing 2 mM phenylmethylsulfonyl fluoride (PMSF) for 4 h. Hyphae were stained with CMAC and visualized under a laser scanning confocal microscopy. White arrows indicate autophagosomes that have not shifted to the vacuole. **(E)** Immunoblot analysis of GFP-FgATG8 degradation in the tested strains. The indicated strains were cultured in liquid CM at 28°C for 48 h and were transferred to liquid MM-N containing 2 mM PMSF for 4 h. Mycelia were collected at the indicated time points from which total protein was extracted and Western blot conducted to check the intensity of GFP-FgATG8 with anti-GFP antibody.

In budding yeast, Vps9 is predominantly cytosolic and partially localizes to endosomes (Paulsel et al., 2013; Bean et al., 2015). In *P. oryzae* and *Fol*, Vps9 colocalize with the early endosome marker Vps21 (Zhu et al., 2018; Li et al., 2019). In order to determine the localization of FgVps9 in *F. graminearum*, we subjected our previously generated complementation strain harboring the GFP-FgVps9 fusion protein to laser scanning confocal microscopy. Our data indicated that GFP-FgVps9 localized to the cytosol and also to some punctate structures (Figure 3A). To verify if the punctate structures were endosomes, we cotransformed the endosome marker mCherry-FgRab51 or FgRab52 with GFP-FgVps9 into PH-1 and found that GFP-FgVps9 colocalized with both mCherry-FgRab51 and mCherry-FgRab52 on the punctate structures in vegetative hyphae (Figure 3A). Thus, we conclude that FgVps9 markedly localizes to the endosomes and is also ubiquitously expressed in the cytosol in *F. graminearum*.

The localization of FgVps9 on the endosomes suggests an important role of the protein in the endocytic process. To verify this hypothesis, we initially checked if the localization of GFP-FgRab51 and GFP-FgRab52 is altered in the Δ*Fgvps9* mutant in comparison with the wild-type PH-1 by laser scanning confocal microscopy. We counted ∼30 hyphal tips, and on average, there were 13-14 GFP-FgRab51- or GFP-FgRab52-labeled punctate structures colocalizing with FM4-64-positive endosomes in each hyphal tip of PH-1. In contrast, only seven to eight GFP-FgRab51 or GFP-FgRab52-labeled punctate endosomes were found in the hyphal tip of the Δ*Fgvps9* mutant (Figure 3B). These results indicate that the number of endosomes was reduced in the Δ*Fgvps9* mutant compared to the wild-type PH-1, suggesting a reduced endocytic process due to *FgVPS9* deletion.

We next monitored the endocytic uptake of the fluorescent dye FM4-64 at different time points in the Δ*Fgvps9* mutant in comparison with the wild-type PH-1. As shown in Figure 3C, FM4-64 was mostly on the plasma membrane after 1 min staining and internalized into the vacuole membrane through endosomes of hyphal cells in the wild-type PH-1 and the complementation strain within 45 min. By contrast, FM4-64 was mainly remained on the plasma membrane and endosomes but not internalized into vacuole membrane in the *FgVPS9-*deficient strain until 45 min (Figure 3C). These results collectively reveal that FgVps9 plays a critical role in the endocytic process.

Autophagy is an ubiquitous and conserved process for recycling and degradation of proteins in eukaryotes and is also found to play an important role in fungal vegetative growth, reproduction, and virulence (Veneault-Fourrey et al., 2006; Kuratsu et al., 2007; Kershaw and Talbot, 2009; Pollack et al., 2009; Lv et al., 2017). Autophagic process can be monitored by observing the delivery of GFP-ATG8-labeled autophagosomes (Cheong and Klionsky, 2008). To determine the role of FgVps9 in autophagy, GFP-FgATG8 fusion protein was transformed into the wild-type PH-1 and the Δ*Fgvps9* mutant strains, respectively. GFP-FgATG8 mainly localized to the punctate structures and vacuole in both PH-1 and the Δ*Fgvps9* mutant when they were grown in nutrient-rich media (liquid CM) for 48 h (Figure 3D, CM). However, GFP-FgATG8 completely localized to the vacuole of the hyphal cells in PH-1, while it remained partially in the punctate structures in the Δ*Fgvps9* mutant when they were shifted to nitrogen starvation media (liquid MM-N media) with 2 mM PMSF and grown for another 4 h (Figure 3D, MM-N). Furthermore, GFP-FgAtg8 immunoblotting assay was also conducted for further confirmation. When grown in liquid CM, the GFP-FgAtg8 level was higher than that of free GFP in PH-1 (Figure 3E), indicating that rich nutrients suppressed the delivery of GFP-FgAtg8 protein into the vacuoles for degradation. By comparison, higher levels of free GFP accumulated in the Δ*Fgvps9* mutant under the same nutrient condition (Figure 3E). On the other hand, when grown in MM-N media, the wild-type PH-1 showed increased proteolysis of the GFP-FgAtg8 and increased GFP/GFP-FgAtg8 ratio (Figure 3E). However, the Δ*Fgvps9* mutant grown in MM-N exhibited the similar GFP/GFP-FgAtg8 ratio to that when it was grown in CM. Collectively, our results suggested that autophagy pathway is impaired in the Δ*Fgvps9* mutant, which demonstrates the critical role of FgVps9 protein in this degradation pathway.

### FgVps9 Is Critical for Virulence

**FIGURE 4:**
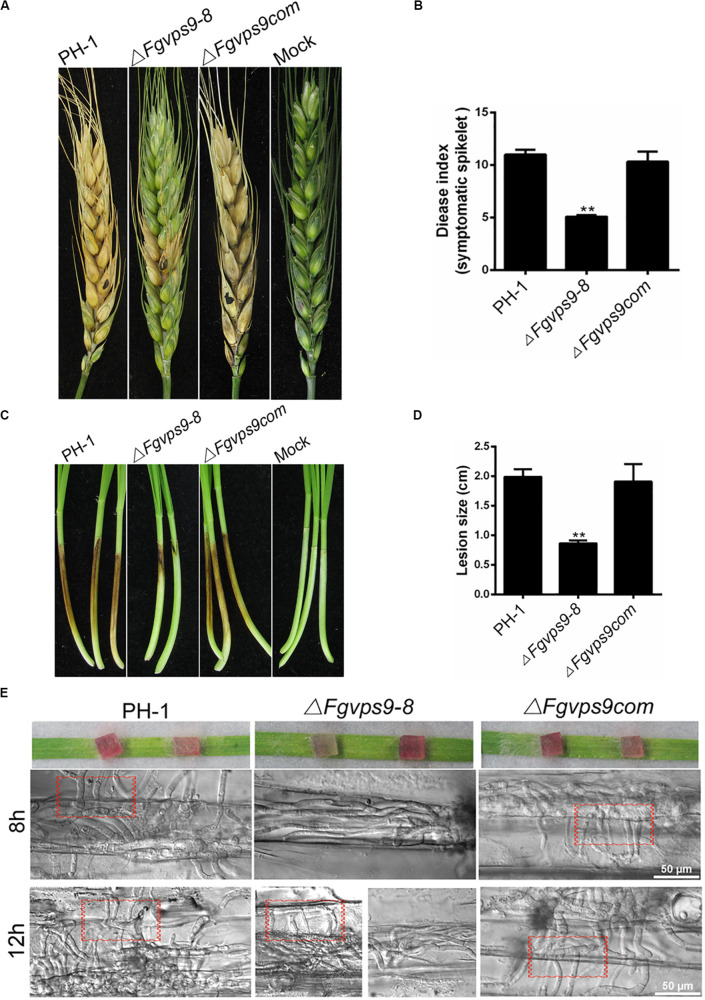
FgVps9 is required for virulence. **(A)** Infection of Δ*Fgvps9* mutant to spikelets was tremendously decreased. Flowering wheat heads were inoculated with mycelia plugs of the wild-type PH-1, Δ*Fgvps9*, and the complementation strain. Photographs were taken at 14 days postinoculation (dpi). Inoculated spikelets were marked by black dot. **(B)** Graphical representation of the disease indices in panel **(A)**. Disease index was evaluated by counting the number of symptomatic spikelets in the corresponding strains after 14 days of inoculation in field. Statistical differences was calculated by multiple *t*-tests from three independent repeats using GraphPad Prism at *p* ≤ 0.05. **(C,D)** Pathogenicity test of the various strains on wheat coleoptiles. The pathogenicity of the Δ*Fgvps9* mutant was significantly reduced. Coleoptiles were inoculated with conidia suspension from the wild-type PH-1, Δ*Fgvps9*, and the complementation strain. Pictures were taken and lesion sizes were measured at 7 dpi. Statistical differences were calculated by multiple *t*-tests with three independent repeats using GraphPad Prism at *p* ≤ 0.05. **(E)** Analysis of cell-to-cell invasion in wounded wheat leaves. The lower epidermis of the detached wheat leaves were inoculated with mycelium plugs from the tested strains. Confocal images were taken at 8 and 12 h postinoculation. Red boxes indicate invasive hyphae that penetrated into surrounding cells.

To gain insight into the role of FgVps9 in pathogenicity of *F. graminearum* to host plants such as wheat, mycelia plugs of the wild-type and mutant strains were inoculated on flowering wheat heads. After 14 days postinoculation (dpi) under moist condition, scab symptoms were examined. Our results showed that the wild-type PH-1 and the complementation strains produced severe head blight symptoms on inoculated wheat heads, while the Δ*Fgvps9* mutant showed reduced virulence and produced little symptom (Figures 4A,B). In addition, we conducted virulence assays on young wheat coleoptiles where similar results were obtained from each of the strains (Figures 4C,D). To confirm the virulence attenuation of the Δ*Fgvps9* mutant, we examined the invasive hyphal growth within the host cell. After incubation with mycelium plugs for 8 h, the growth of invasive hyphae from the Δ*Fgvps9* mutant was limited within one cell, while those from PH-1 and complementation strains had penetrated into the surrounding cells, even when the incubation period was extended to 12 h; majority of the infectious hyphae from the Δ*Fgvps9* mutant remained in one cell (Figure 4E, red box). These findings suggested that FgVps9 is crucial for plant infection of *F. graminearum*, and the virulence attenuation of the Δ*Fgvps9* mutant is largely due to a defect in cell-to-cell movement by the infectious hyphae.

### FgVps9 Is Indispensable for DON Production

**FIGURE 5:**
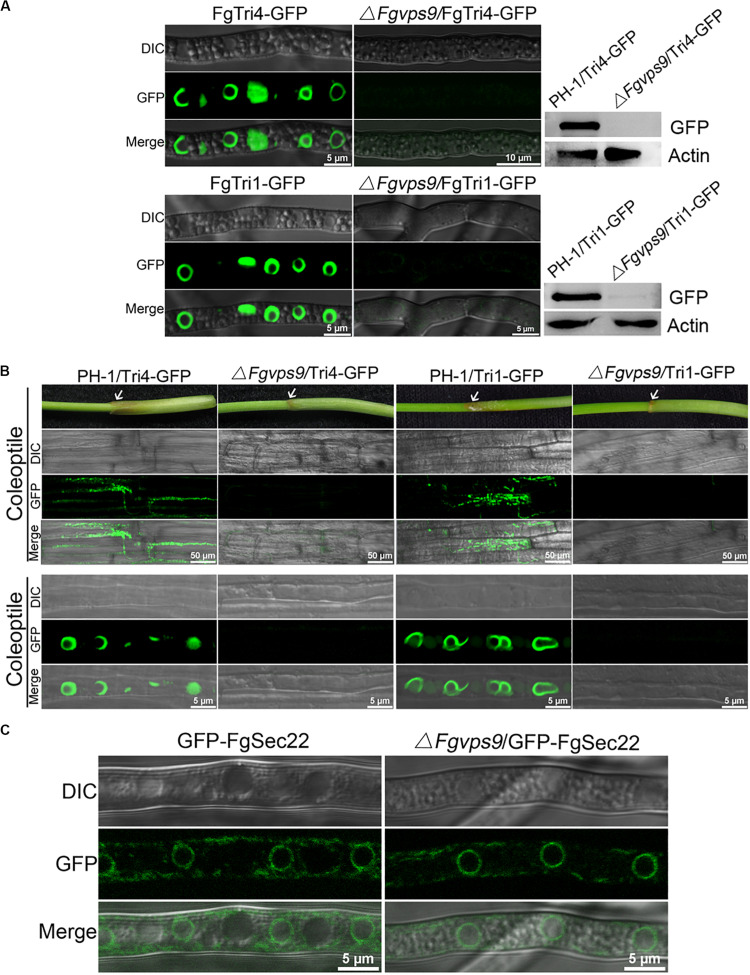
FgVps9 is pivotal for deoxynivalenol (DON) biosynthesis. **(A)** FgTri4- and FgTri1-labeled toxisomes formation in the indicated strains. Toxisome formation was not observed in the Δ*Fgvps9* mutant. Toxisome was visualized by laser scanning confocal microscopy after the indicated strains were cultivated in liquid trichothecene biosynthesis inducing (TBI) media at 28°C under dark condition for 72 h. The intensity of FgTri4-GFP and FgTri1-GFP proteins in the indicated strains were quantified by immunoblot assay using the antigreen fluorescent protein (anti-GFP) antibody (right panel). The protein Actin was used as a reference in the Western blot assay (right panel). **(B)** Toxisome formation in the invasive hyphae. Toxisomes in the invasive hyphae were examined 3 days after conidia from the tested strain-inoculated coleoptiles. Toxisomes were displayed by marker protein FgTri4-GFP and FgTri1-GFP. Images were captured under laser scanning confocal microscopy. White arrows indicate the sites of conidia inoculation on the coleoptiles. **(C)** Localization of GFP-FgSec22 [endoplasmic reticulum (ER) biomarker] in the Δ*Fgvps9* mutant. The tested strains were cultivated in liquid TBI media at 28°C under dark condition. Images were captured after 2 days.

DON is a well-documented virulence factor in the pathogenicity of *F. graminearum* on wheat (Proctor et al., 1995; Desjardins, 1996). Therefore, we investigated and compared the levels of DON produced by the wild-type PH-1 and the Δ*Fgvps9* mutant. The Δ*Fgvps9* mutant produced much lower levels of DON compared to PH-1 and the complementation strain after cultivating these fungal strains in liquid trichothecene biosynthesis-inducing (TBI) media for 7 days (Table 1). We then determined the transcription levels of the transcription factor *TRI6* and the three trichothecene biosynthesis-related genes *TRI5*, *TRI1*, and *TRI4* (Seong et al., 2009) by quantitative reverse transcription PCR (qRT-PCR) analysis. The results showed that the expression levels of *TRI6*, *TRI5*, *TRI4*, and *TRI1* in the Δ*Fgvps9* mutant were extremely lower when compared to those in the wild-type PH-1 (Table 2). To further confirm these results, we investigated the expressions of the toxisome-localized proteins FgTri1 and FgTri4 (Boenisch et al., 2017; Tang et al., 2018) by tagging them with GFP in both PH-1 and the Δ*Fgvps9* mutant and subjecting them to a fluorescence confocal microscope. As shown in Figure 5A, FgTri4- and FgTri1-labeled toxisomes were barely visible in the Δ*Fgvps9* mutant, but they were readily identified in the wild-type PH-1 strain. Consistently, the protein levels of the FgTri4-GFP and FgTri1-GFP proteins were barely detectable in the Δ*Fgvps9* mutant compared to those in the wild-type PH-1 (Figure 5A, right panel). Moreover, upon inoculation of conidia on coleoptiles, toxisomes were formed in the invasive hyphae of PH-1 but not of Δ*Fgvps9* mutant (Figure 5B). Boenisch et al. (2017) previously showed that endoplasmic reticulum (ER) was reorganized in TBI media, and Tri4 and Tri1 were localized to this expanded structure. Sec22 is a typical ER marker in yeast, *Aspergillus oryzae*, and animals (Kuratsu et al., 2007; Petkovic et al., 2014; Zhao et al., 2015). We thus monitored the ER structure by checking the localization of GFP-FgSec22 in the Δ*Fgvps9* mutant and PH-1. Our results showed normal and similar ER structures in both the Δ*Fgvps9* mutant and the wild-type PH-1 (Figure 5C), indicating that FgVps9 plays a specific and crucial role in Tri4/Tri1-associated toxisome formation but not in the general ER structure. Collectively, these findings reveal that FgVps9 participates in DON production by regulating the expression of *TRI* genes (and hence toxisome biogenesis) rather than altering the ER structure.

**TABLE 1:** Deoxynivalenol (DON) production by Δ*Fgvps9* deletion mutant.

**Strain**	**DON (μg/g dry weight mycelia)**
PH-1	152.46 ± 15.07
△Fgvps9-8	0.92 ± 0.28*
△Fgvps9com	146.95 ± 2.30

*DON was extracted from the mycelia of the indicated strains incubated in liquid TBI under dark condition for 7 days. ±SD was calculated from three independent replicates, and asterisk indicates significant differences compared to the wild-type PH-1.*

**TABLE 2:** Transcription levels of *TRI* genes in Δ*Fgvps9* mutant.

**Strain**	**Transcription level**			
	***TRI1***	***TRI4***	***TRI5***	***TRI6***
PH-1	1	1	1	1
△Fgvps9-8	0.40 × 10^–3^ ±	0.55 × 10^–3^ ±	0.83 × 10^–3^ ±	0.48 × 10^–2^ ±
	0.48 × 10^–4^*	0.26 × 10^–3^*	0.37 × 10^–3^*	0.18 × 10^–2^*

*Relative expression level was measured after the indicated strains were incubated in liquid TBI media at 28°C under the dark condition for 3 days. ±SD was calculated from three independent replicates, and asterisks indicate significant differences compared to the wild-type PH-1.*

### FgRab51 and FgRab52 Also Play a Critical Role in DON Biosynthesis

**FIGURE 6:**
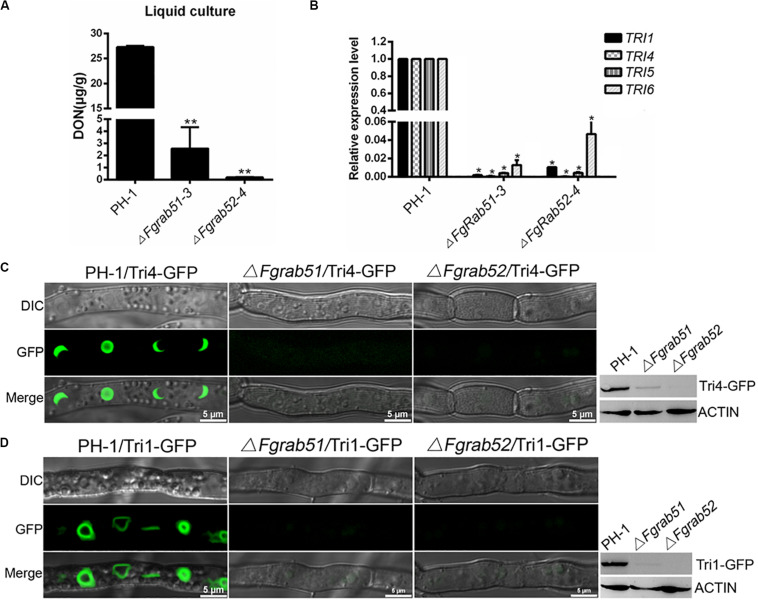
FgRab5 is involved in deoxynivalenol (DON) biosynthesis. **(A)** The DON production of the Δ*Fgrab5* mutants. DON was extracted from mycelia of the indicated strains incubated in liquid trichothecene biosynthesis inducing (TBI) media under dark condition for 7 days. Asterisk represent significant differences compared to the wild-type PH-1. **(B)** Transcription level of *TRI* genes in the Δ*Fgrab5* mutants. Relative expression level was measured after the indicated strains incubated in liquid TBI media at 28°C under the dark condition for 3 days. **(C,D)** Toxisome FgTri4- and FgTri1-labeled was not observed in the Δ*Fgrab5* mutant. Toxisome was visualized by a laser scanning confocal microscopy after the indicated strains were cultivated in liquid TBI media at 28°C under the dark condition for 72 h. The intensity of FgTri4-GFP and FgTri1-GFP in the indicated strains were identified by immunoblot assay using the antigreen fluorescent protein (anti-GFP) antibody (right panel). The protein Actin was used as a reference in the Western blot assay (right panel).

As described above, FgVps9 may function as a GEF for activation of FgRab51 and FgRab52. Next, we determined whether FgRab51 and FgRab52 play any role in DON biosynthesis by measuring DON levels in the Δ*Fgrab51* and Δ*Fgrab52* mutants in liquid TBI media. As shown in Figure 6A, DON levels drastically decreased in both mutants in comparison to the wild-type PH-1. The expression levels of *TRI6*, *TRI5*, *TRI4*, and *TRI1* genes were also downregulated in the mutants compared to those in the wild-type PH-1 (Figure 6B). Similar to the Δ*Fgvps9* results, FgTri4- and FgTri1-labeled toxisomes were not detected in the Δ*Fgrab51* and Δ*Fgrab52* mutants but were readily visible in the wild-type PH-1 strain (Figures 6C,D). Taken together, these results demonstrate that FgRab51 and FgRab52 also play an indispensable role in DON biosynthesis and toxisome biogenesis in *F. graminearum*, like FgVps9.

### The Vps9 Domain of FgVps9 Is Indispensable for Its Biological Function

**FIGURE 7:**
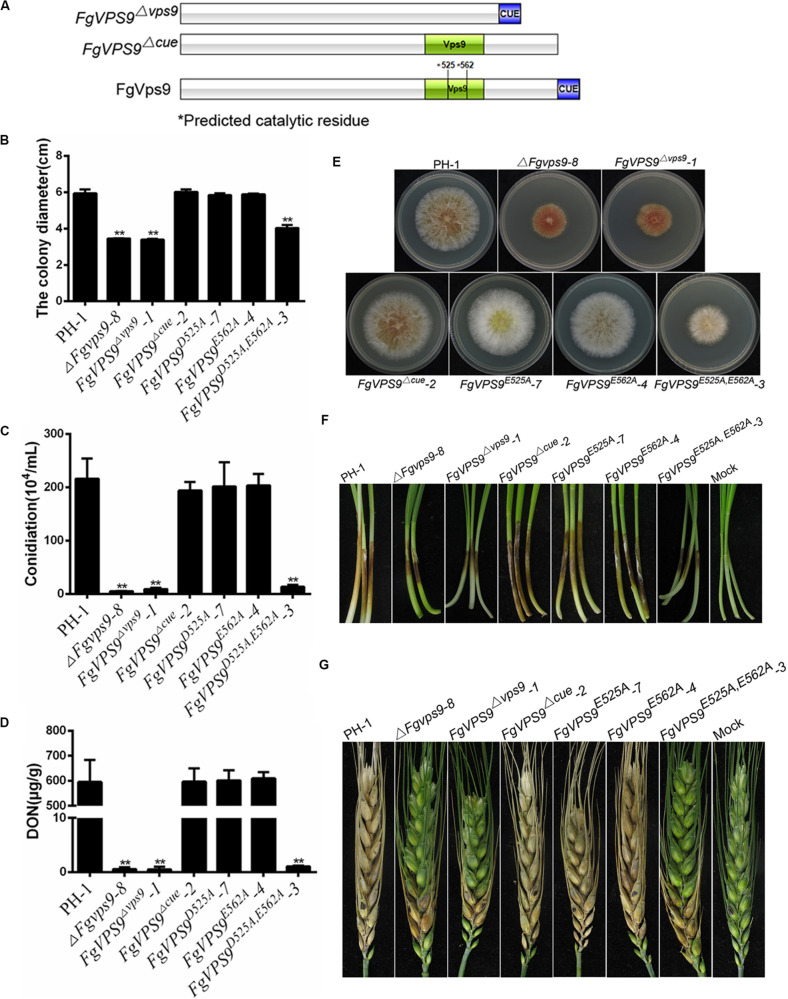
The conserved Vps9 domain of FgVps9 is required for the biological functions of the whole protein. **(A)** Schematic diagram and strategy of domain deletions and point mutations of FgVps9. *525 (Asp525Ala, D525A), *562 (Glu562Ala, E562A). **(B)** Average colony diameters of the indicated strains incubated on complete media (CM) for 3 days. Statistical differences were calculated by multiple *t*-tests from three independent repeats using GraphPad Prism at *p* ≤ 0.05. **(C)** Conidiation assay. The conidia were counted after inoculation and incubation of the indicated strains in CMC media for 3 days. Statistical differences were calculated by multiple *t*-tests from three independent repeats using GraphPad Prism at *p* ≤ 0.05. **(D)** Deoxynivalenol (DON) production assay. DON was extracted from mycelia of the indicated strains incubated in trichothecene biosynthesis inducing (TBI) media for 7 days. Asterisks indicate significant differences at *p* ≤ 0.05. **(E)** The vegetative growth of the indicated strains cultured on CM at 28°C for 3 days. **(F)** Pathogenicity assay of various strains to wheat coleoptiles. Wheat coleoptiles were inoculated with conidia suspension from the indicated strains, and pictures were taken at 7 dpi. **(G)** Pathogenicity of the indicated strains on flowering wheat heads. The flowering wheat heads were inoculated with mycelia plugs of the indicated strains. Photographs were taken at 14 dpi. Inoculated spikelets were marked by black dots.

**FIGURE 8:**
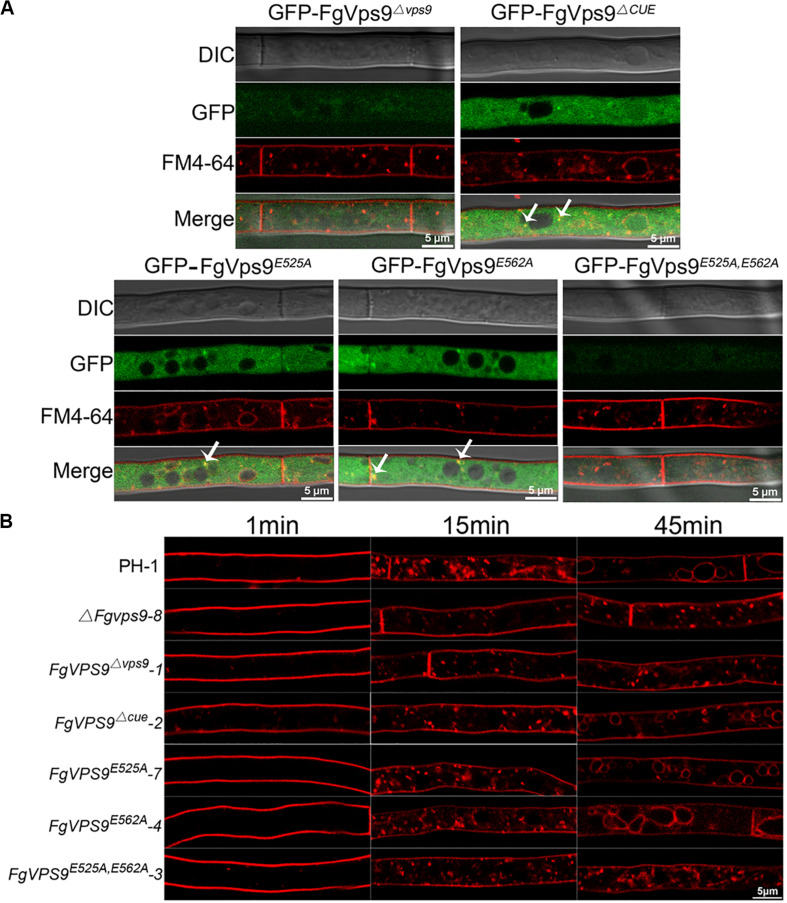
The Vps9 domain of FgVps9 is required for endosomal localization and endocytosis in *F. graminearum*. **(A)** Localization of the mutated GFP-FgVps9 in the indicated mutant strains. Each strain was cultivated in liquid complete media (CM) for 24 h, then stained with FM4-64 and observed under a fluorescence confocal microscope. White arrows indicate colocalization. **(B)** FM4-64 internalization assay in the indicated strains. Hyphae of the various strains were incubated in liquid CM at 28°C for 24 h, then stained with FM4-64 and observed under a fluorescence confocal microscope at different time points.

In the budding yeast, the conserved residues Asp_251_ and Glu_288_ for GEF activity in the Vps9 domain play a crucial role in growth and carboxypeptidase Y (CPY) and carboxypeptidase S (Cps1) sorting (Shideler et al., 2015). In order to understand the functions of Vps9 domain, CUE domain, and the GEF activity of FgVps9, we generated domain deletion (*FgVPS9^Δvps9^*, *FgVPS9^ΔCUE^*) and point mutation (*FgVPS9^D525A^*, *FgVPS9^D562A^*, and *FgVPS9^D525A,D562A^*) (Figure 7A) constructs and transformed them into the Δ*Fgvps9* mutant. The phenotypes of the resulting transformants were systematically analyzed. As shown in Figure 7, *FgVPS9^Δvps9^* and the double-point mutant *FgVPS9^D525A,D562A^* displayed similar phenotypes to the Δ*Fgvps9* mutant in vegetative growth, sporulation, DON production, and pathogenicity on wheat, whereas *FgVPS9^ΔCUE^*, *FgVPS9^D525A^*, and *FgVPS9^D562A^* showed no significant phenotypic differences from the wild-type PH-1 (Figures 7B–G). Moreover, subcellular localization analysis indicated that GFP-*FgVPS9^Δ*vps*9^* and GFP-*FgVPS9^D525A,D562A^* failed to associate with the endosomes, while GFP-*FgVPS9^ΔCUE^*, GFP-*FgVPS9^D525A^*, and GFP-*FgVPS9^D562A^* were found to colocalize with the FM4-64-labeled endosomes (Figure 8A). Furthermore, we monitored the internalization of FM4-64 in these strains. Similar to the Δ*Fgvps9* mutant, *FgVPS9^Δ*vps*9^* and *FgVPS9^D525A,D562A^* showed defective endocytic trafficking to the vacuole with FM4-64 accumulation on the plasma membrane and endosomes after 45 min incubation. By contrast, FM4-64 was already transported to the vacuole membrane in *FgVPS9*^Δ*CUE*^, *FgVPS9^D525A^*, and *FgVPS9^D562A^* mutants (Figure 8B). These results collectively suggested that Vps9 domain and the GEF activity are essential for the growth, conidiation, DON production, pathogenicity, and endocytosis in *F. graminearum*.

## Discussion

Rab GTPases are key regulators of vesicle-mediated membrane trafficking system (endocytosis and exocytosis) in all eukaryotic organisms (Hutagalung and Novick, 2011). Rabs act as molecular switches by conformational exchange between inactivate GDP-bound and active GTP-bound states, while GEFs facilitate GTP binding to the Rab proteins and make them to be active state (Barr and Lambright, 2010). Previous studies have determined that Vps9 functions as GEF of Vps21 (Rab5) in the budding yeast and phytopathogenic fungi *P. oryzae* and *Fol* (Hama et al., 1999; Zhu et al., 2018; Li et al., 2019). However, the physiological and pathological roles of Vps9 are still unknown in *F. graminearum*. In this study, we have identified the Vps9 homolog FgVps9 in the plant pathogenic fungi *F. graminearum* and found that FgVps9 functions as GEF of FgRab5. FgVps9 specifically interacts with FgRab51DN and FgRab52DN and colocalized with FgRab5 to the early endosomes. Moreover, FgVps9 plays a vital and direct role in endocytosis, which has significant impact on the fungal growth, conidiation, autophagy, virulence, and DON production.

*Fusarium graminearum* can infect a variety of cereal crops and produce trichothecene mycotoxin such as DON in the infested grains, making the grains harmful for human and livestock consumption (Goswami and Kistler, 2004). Endocytosis is crucial for the uptake and signal transduction of extracellular substances and plasma-membrane-associated proteins (Sorkin and von Zastrow, 2009; Goode et al., 2015) and is evolutionarily conserved. Even though the relationship between endocytosis and DON production is still unclear, deletion of some endocytosis-related genes perturbed DON production in *F. graminearum*. For example, the FgRab7 GEF FgMon1, the HOPS complex subunit FgVps39, and the soluble *N*-ethylmaleimide-sensitive factor attachment protein receptors (SNAREs) FgVam7 are involved in the endocytic pathway, and DON production is significantly decreased in their respective deletion mutants (Li et al., 2015; Li B. et al., 2017; Zhang et al., 2016). Furthermore, Zheng et al. (2015) found that deletion of *FgRAB51* and *FgRAB52* significantly reduced DON production in the infected grains. In our study, we found that endocytosis is delayed and DON production is significantly declined in the *FgVPS9* deletion mutant. In comparison with the wild-type strain, the vegetative growth of *FgVPS9* deletion mutant decreases by ∼50%, while DON production per gram dry weight mycelia decreases by 99%, indicating that the growth defect of the Δ*Fgvps9* mutant is not the main reason for the reduction in DON biosynthesis. Further qRT-PCR and localization analysis established that FgVps9 modulates DON biosynthesis by regulating the expression of trichothecene biosynthesis-related genes *TRI6*, *TRI5*, *TRI1*, and *TRI4*. Because FgVps9 acts as a GEF of FgRab5, we also investigated and found that the relative expression level of *TRI6*, *TRI5*, *TRI1*, and *TRI4* and DON production drastically dropped, and the localization of FgTri1 and FgTri4 in toxisomes is barely detected in the *FgRAB51* and *FgRAB52* deletion mutants, suggesting that FgVps9 and FgRab5 collaboratively modulate DON biosynthesis by regulating the expression of the trichothecene biosynthesis-related genes. These data collectively demonstrate that endocytosis plays an essential role in DON production in *F. graminearum*. In addition, studies have shown that vesicles and vacuoles contain some enzymes associated with secondary metabolism in fungi and plants, including enzymes involved in the biosynthesis of flavonoids, alkaloids, the β-lactam antibiotic penicillin, the non-ribosomal peptide cyclosporine, and the polyketide aflatoxin (Hoppert et al., 2001; Lee et al., 2004; Hong and Linz, 2008; Ziegler and Facchini, 2008; Chanda et al., 2009). In the filamentous fungus *Aspergillus parasiticus*, proteomic and biochemical analyses suggested that most enzymes involved in aflatoxin formation are stored in endosomes, transport vesicles, and vacuoles (Chanda et al., 2009; Linz et al., 2012). Previous studies have indicated that FgTri1 and FgTri4 are mainly localized to the toxisomes (the expanded ER) and partially to the motile vesicles; FgTri12 is localized to the plasma membrane, vesicles, and vacuoles in DON biosynthesis-inducing condition, while these vesicles are similar to the endosomes (Menke et al., 2012, 2013; Kistler and Broz, 2015). Meanwhile, in our study, we discovered that FgVps9 functions as a GEF of FgRab5 and colocalize with FgRab5 on the early endosomes. These data suggest that FgVps9 and FgRab5 may participate in endosome and vesicular transport; deletion of these genes may abrogate storage and transport of enzymes related to the synthesis of mycotoxins in endosomes and consequently disrupt the biosynthesis and storage of trichothecene mycotoxins in these organelles. To our knowledge, this is the first evidence to establish the relationship between FgVps9-FgRab5 and DON biosynthesis in fungi. Proteomic and biochemical analyses will be necessary for determining the distribution of enzymes related to mycotoxins biosynthesis in FgVps9 and FgRab5-labeled endosomes in further studies.

In the phytopathogenic fungus *Ustilago maydis*, the endocytosis-associated protein Yup1 is localized to the early endosomes and is critical for the early stage of pathogenic development (Fuchs et al., 2006). The SNARE proteins MoVam7, MoSyn8, and FgVam7 in *P. oryzae* and *F. graminearum* have been identified to participate in endocytosis and pathogenicity, respectively (Dou et al., 2011; Qi et al., 2016; Zhang et al., 2016). Deletion of the endocytosis-related gene *RAB5* in *P. oryzae* and *F. graminearum* results in growth defect and complete loss of the infection to plants (Zheng et al., 2015; Yang et al., 2017). Moreover, Vps9, a Vps21 GEF, colocalizes with the early endosome marker Vps21 and plays an important role in vegetative growth, endocytosis, and pathogenicity in the rice blast fungus *P. oryzae* and the tomato pathogen *Fol* (Zhu et al., 2018; Li et al., 2019). These results collectively reveal that endocytosis is closely associated with the growth and pathogenicity of the plant fungal pathogens. In our study, deletion *FgVPS9* also impaired the fungal radial growth, internalization of FM4-64, the fungal virulence on wheat, and cell-to-cell movement of the invasive hyphae, suggesting that the disruption of endocytosis in the mutant results in the observed defects in growth and pathogenicity of *F. graminearum*. Moreover, previous studies have demonstrated that DON is a virulence factor, and the virulence of trichothecene-non-producing mutants on wheat is reduced in *F. graminearum* (Proctor et al., 1995; Desjardins, 1996). In our study, DON is also significantly reduced in the *FgVPS9* deletion mutant, indicating that the reduced virulence of the Δ*Fgvps9* mutant on wheat may partially result from the reduction in DON production. These phenotypes of the Δ*Fgvps9* mutant are similar to that of the reported *FgRAB5* deletion mutants (and the present study presented FgVps9 as a GEF of FgRab5), indicating that the endocytosis-related proteins FgVps9 and FgRab5 cooperatively regulate the growth and virulence of *F. graminearum*.

Autophagy, a non-selective degradation pathway responsible for the turnover of proteins, organelles, and membranes, is conserved from yeast to human (Abeliovich and Klionsky, 2001; Alva et al., 2004; Mizushima, 2007). Many studies have demonstrated that autophagy plays an important role in radial growth, asexual/sexual reproduction, environmental stresses, and plant infection in phytopathogenic fungi (Veneault-Fourrey et al., 2006; Liu et al., 2007; Asakura et al., 2009; Kershaw and Talbot, 2009; Pollack et al., 2009; Kikuma et al., 2014). Deletion of autophagy-related genes also hindered DON production and virulence in *F. graminearum* (Nguyen et al., 2011; Josefsen et al., 2012; Lv et al., 2017). In recent years, autophagy has been found to be associated with endocytosis, and same proteins function in both processes. Vesicle-trafficking-related proteins Rab5/Vps21, FgRab7, and its GEF FgMon1 are involved in both autophagic pathway and endocytosis in *S. cerevisiae* and *F. graminearum*, respectively (Singer-Kruger et al., 1994; Chen et al., 2014; Li et al., 2015; Zheng et al., 2015, 2018b). In addition, deletion of *VPS9* in *P. oryzae* and *Fol* seriously disrupted fungal growth and pathogenicity by blocking autophagic and endocytic processes (Zhu et al., 2018; Li et al., 2019). In our study, *FgVPS9* deletion mutant also shows reduced vegetative growth, endocytosis, virulence, and DON production, while autophagy is only partially blocked in Δ*Fgvps9* mutant with most autophagosomes labeled by GFP-ATG8 fused to vacuoles in liquid MM-N media. However, in *P. oryzae* and *Fol*, remarkably less fluorescence signals of GFP-ATG8 and few autophagosomes were examined in the vacuoles of the Δ*Povps9* and Δ*Folvps9* mutants when comparing with those of wild-type strains, suggesting that autophagy is seriously blocked in Δ*Povps9* and Δ*Folvps9* mutants (Zhu et al., 2018; Li et al., 2019). Collectively, these data indicated that, similar with PoVps9 and FolVps9, FgVps9 functions in both endocytosis and autophagy processes, which involved in fungal growth, virulence, and DON production; however, the role of FgVps9 on autophagy is not as important as its homologs in *P. oryzae* and *Fol*.

Structure analysis has revealed that Vps9 domain and the two GEF activity sites of Vps9 protein, rather than CUE domain, play an indispensable role in vegetative growth and internalization of carboxypeptidase S (CPS) in the budding yeast (Shideler et al., 2015). Here, our data have shown that Vps9 domain, rather than CUE domain, in FgVps9 regulates the endosomal localization and physiological functions of the entire protein in *F. graminearum*. This result is consistent with that observed in *Fol* (Li et al., 2019), indicating that the function of Vps9 domain is pleiotropic in fungi. We also found that the two GEF activity sites in the FgVps9 domain play an essential role in *F. graminearum*. The double-point mutation *FgVPS9^D525A,D562A^* showed similar phenotypic defects to FgVps9 domain deletion mutant in the radial growth, conidiation, endocytosis, plant infection, and DON production, while the single-point mutations *FgVPS9^D525A^* and *FgVPS9^D562A^* have no significant difference compared to the wild-type PH-1 strain in all the analyzed phenotypes. This suggests that the GEF activity is also indispensable for the functions of FgVps9 in *F. graminearum*.

In conclusion, we have herein identified and characterized FgVps9, a vacuolar protein sorting-associated protein, which shows similar biological functions to FgRab5 in *F. graminearum*. Moreover, it functions as GEF of FgRab51 and FgRab52 by specifically interacting with their GDP-bound forms and thus plays a critical role in the radial growth, asexual development, endocytosis and autophagy, plant infection, and DON production by cooperating with FgRab5 in *F. graminearum*. Our results have also demonstrated that, in *F. graminearum*, endocytosis is, to some extent, associated with DON biosynthesis. The reduced DON production and virulence of the Δ*Fgvps9* mutant could have resulted from functional deficiency of FgRab5 due to *FgVPS9* deletion in *F. graminearum*. Future studies will concentrate on identification of more endocytosis-related proteins and molecular mechanism of how these proteins regulate DON biosynthesis.

## Data Availability Statement

All datasets presented in this study are included in the article/Supplementary Material.

## Author Contributions

JZ conceived and designed the experiments. CY, JL, XC, XZ, and DL performed the experiments. CY wrote the manuscript. JZ, ZW, GL, YA, WZ, and YY revised and approved the manuscript. All authors contributed to the article and approved the submitted version.

## Conflict of Interest

The authors declare that the research was conducted in the absence of any commercial or financial relationships that could be construed as a potential conflict of interest.

## Footnotes

1 http://smart.embl-heidelberg.de/

2 http://fungidb.org/fungidb/

## Supplementary Material

FIGURE S1 Domains architecture and phylogenetic analysis of FgVps9 proteins. **(A)** Schematic diagram showing the domains present in FgVps9. Vps9 (Vacuolar sorting protein 9) protein in *F. graminearum* was identified using amino acids alignment of yeast homologs and the SMART program (http://smart.embl-heidelberg.de/). The protein contains two domains: Vps9 and CUE domains. **(B)** Phylogenetic tree of Vps9 proteins in *S. cerevisiae*, *P. oryzae*, *F. graminearum*, and *Fol*. The tree was constructed by neighbor-joining method with 1,000 bootstrap replicates in ClustalW and MEGA5.2.

FIGURE S2 Southern blot analysis of the indicated gene deletion mutants. **(A)** The split-marker strategy for targeted genes deletion. The indicated strains were grown in liquid CM at 28°C for 48 h. Genomic DNA was extracted from these strains and digested with the restriction enzymes. Primers presented in Supplementary Table S1 and probes were used for mutant screening and identification. **(B)** Deletion strategy for *FgVPS9* and its Southern blot confirmation. A PCR fragment was amplified from FgVPS9 (SCB64083.1) with primer pairs AF/AR and used as a probe for Southern blot assay. Genomic DNAs from wild type and the mutant were digested with *Eco*RV (E) at 37°C for 48 h. The blotting assay displayed a 5.334 kb band in the wild-type PH-1 and a 4.027 kb band in the *FgVPS9* deletion mutant.

TABLE S1 List of primer pairs used in this study.
