# Supplementary material for: FgVps9, a Rab5 GEF, Is Critical for DON Biosynthesis and Pathogenicity in Fusarium graminearum
Source: Front Microbiol. 2020 Aug 4;11:1714. doi: 10.3389/fmicb.2020.01714 (PMC7418515; doi:10.3389/fmicb.2020.01714)
Supplement: FIGURE S1 — Domains architecture and phylogenetic analysis of FgVps9 proteins. (A) Schematic diagram showing the domains present in FgVps9. Vps9 (Vacuolar sorting protein 9) protein in F. graminearum was identified using amino acids alignment of yeast homologs and the SMART program (http://smart.embl-heidelberg.de/). The protein contains two domains: Vps9 and CUE domains. (B) Phylogenetic tree of Vps9 proteins in S. cerevisiae, P. oryzae, F. graminearum, and Fol. The tree was constructed by neighbor-joining method with 1,000 bootstrap replicates in ClustalW and MEGA5.2. [file Table_1.doc]

**FgVps9, a Rab5 GEF, is critical for DON biosynthesis and pathogenicity in *Fusarium graminearum***

Chengdong Yang^1,2^, Jingjing Li^1,2^, Xin Chen^1,2^, Xingzhi Zhang^1,2^, Danhua Liao^1,2^, Yingzi Yun^2^, Wenhui Zheng^2^, Yakubu Saddeeq Abubakar^3^, Guangpu Li^4^, Zonghua Wang^1,2,5^ and Jie Zhou^1,2＊^

1 Fujian Province Key Laboratory of Pathogenic Fungi and Mycotoxins and College of Life Sciences, Fujian Agriculture and Forestry University, Fuzhou, 350002,China.

2 State Key Laboratory of Ecological Pest Control for Fujian and Taiwan Crops, Fujian Agriculture and Forestry University, Fuzhou, 350002, China.

3 Department of Biochemistry, Ahmadu Bello University, Zaria-Nigeria.

4 Department of Biochemistry and Molecular Biology, University of Oklahoma Health Sciences Center, Oklahoma City, Oklahoma, United States of America.

5 Institute of Oceanography, Minjiang University, Fuzhou, 350108, China.

＊Authors for Correspondence: Dr. Jie Zhou

E-mail: jiezhou@fafu.edu.cn

**Running title:** Vps9 in *Fusarium graminearum*

**Key words: FgVps9, guanine nucleotide exchange factor (GEF), endocytosis, Pathogenicity, DON, *Fusarium graminearum***

**Supplementary material**

**
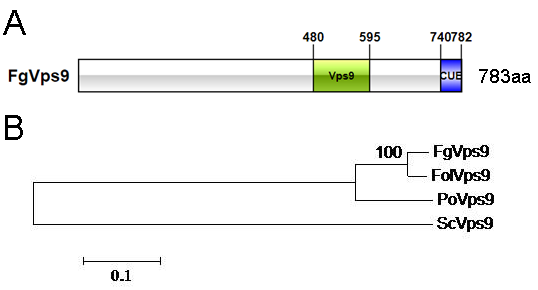
**

**FIGURE S1∣Domains architecture and phylogenetic analysis of FgVps9 proteins.**

**(A)** Schematic diagram showing the domains present in FgVps9. Vps9 (Vacuolar sorting protein 9) protein in *F. graminearum* was identified using amino acids alignment of yeast homologs and the SMART program (<http://smart.embl-heidelberg.de/>). The protein contains two domains: Vps9 and CUE domains.

**(B)** Phylogenetic tree of Vps9 proteins in *S. cerevisiae*, *P. oryzae*, *F. graminearum* and *Fol*. The tree was constructed by neighbor-joining method with 1,000 bootstrap replicates in ClustalW and MEGA5.2.


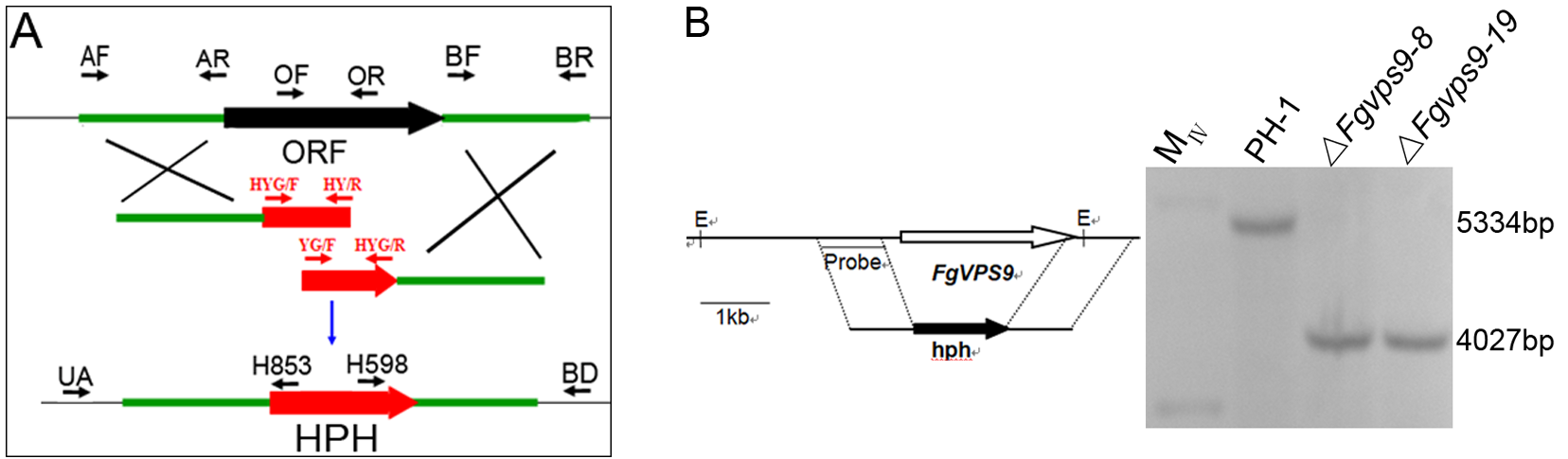


**FIGURE S2∣Southern blot analysis of the indicated gene deletion mutants.**

**(A)** The split-marker strategy for targeted genes deletion. The indicated strains were grown in liquid CM at 28℃ for 48 h. Genomic DNA was extracted from these strains and digested with the restriction enzymes. Primers presented in Table S1 and probes were used for mutant screening and identification.

**(B)** Deletion strategy for *FgVPS9* and its Southern blot confirmation. A PCR fragment was amplified from FgVPS9 (SCB64083.1) with primer pairs AF/AR and used as a probe for Southern blot assay. Genomic DNAs from wild type and the mutant were digested with *Eco*RV (E) at 37℃ for 48 h. The blotting assay displayed a 5.334 kb band in the wild-type PH-1 and a 4.027 kb band in the *FgVPS9* deletion mutant.

**Table S1** List of primer pairs used in this study

| Primers | （5’ → 3’）  Sequences | Function |
| --- | --- | --- |
| *FgVPS9* AF | CCTTGTAGGCTTGGTAGA | *FgVPS9* deletion |
| *FgVPS9* AR | TTGACCTCCACTAGCTCCAGCCAAGCCAAGTTGTCGGGTCAGAGT |
| *FgVPS9* BF | GAATAGAGTAGATGCCGACCGCGGGTTGCATGTTCCCTGACCTTGAC | *FgVPS9* deletion |
| *FgVPS9* BR | GTAACAGCCTGATTGCGTGA |
| *FgVPS9* OF | CGATAGGGAAGTCGGGTAC | *FgVPS9* deletion |
| *FgVPS9* OR | GCTCCAGGAATCTGTGAAA |
| *FgVPS9* PF | AGGGAACAAAAGCTGGGTACCCAGGCCGTTCATCAATGACT | *FgVPS9* native promoter |
| *FgVPS9* PR | TCCTCGCCCTTGCTCACCATACAAAGCTTTCGAAGAGTTG |
| *GFP* F | ATGGTGAGCAAGGGCGAGGA | *GFP* |
| *GFP* R | CTTGTACAGCTCGTCCATGC |
| *FgVPS9* GF | GCATGGACGAGCTGTACAAGATGTCTCCGCCGGAGCAATC | *FgVPS9 ORF* |
| *FgVPS9* GR | CAGTAACGTTAAGTGGATCCTCATGTTGACAGAGCGAGGC |
| *FgVPS9* GF | GCATGGACGAGCTGTACAAGATGTCTCCGCCGGAGCAATC | For  *FgVPS9^D525A^* construction |
| *FgVPS9^D525A^* R1 | ATAAAGGAAGCAGCCGAAGAATC |
| | *FgVPS9^D525A^* F2 | GATTCTTCGGCTGCTTCCTTTAT | | --- | --- | | GATTCTTCGGCTGCTTCCTTTAT |
| *FgVPS9* GR | CAGTAACGTTAAGTGGATCCTCATGTTGACAGAGCGAGGC |
| *FgVPS9* GF | GCATGGACGAGCTGTACAAGATGTCTCCGCCGGAGCAATC | For  *FgVPS9^E562A^* construction |
| *FgVPS9^E562A^* R1 | AGCTGCACCCCCAAGCTTTTCCT |
| *FgVPS9^E562A^* F2 | AGGAAAAGCTTGGGGGTGCAGCT |
| *FgVPS9* GR | CAGTAACGTTAAGTGGATCCTCATGTTGACAGAGCGAGGC |
| *FgVPS9* GF | GCATGGACGAGCTGTACAAGATGTCTCCGCCGGAGCAATC | For  *FgVPS9^△VPS9^* construction |
| *FgVPS9^△VPS9^* R1 | GCCGAGACGGCCTCTTCCACTCGACGGCCACTCTCACC |
| *FgVPS9^△VPS9^* F2 | GTGGAAGAGGCCGTCTCG |
| *FgVPS9* GR | CAGTAACGTTAAGTGGATCCTCATGTTGACAGAGCGAGGC |
| *FgVPS9* GF | GCATGGACGAGCTGTACAAGATGTCTCCGCCGGAGCAATC | For  *FgVPS9^△CUE^* construction |
| *FgVPS9^△CUE^* R | GCCCTTGCTCACCATAAGCTTTGTTGCACGCGTAAGTCTTT |
| *FgRab51* F  *FgRab51DN/CA* F | CTGATCTCAGAGGAGGACCTGCATATGGCCGATTCCACCAACGC | For Y2H |
| *FgRab51* R  *FgRab51DN/CA* R | CGCTGCAGGTCGACGGATCCCCGGGAACTAGGAAGCGGAACTATCCT |
| *FgRab52* F  *FgRab52DN/CA* F | CTGATCTCAGAGGAGGACCTGCATATGGCTTCCCGACAACCTCC | For Y2H |
| *FgRab52 R*  *FgRab52DN/CA* R | CGCTGCAGGTCGACGGATCCCCGGGAATTAGGAACTGGAAGGGCCGC |
| *FgVPS9* ADF | GACGTACCAGATTACGCTCATATGTCTCCGCCGGAGCAATC | For Y2H |
| *FgVPS9* ADR | TATCGATGCCCACCCGGGTGGAATCATGTTGACAGAGCGAGGC |
| FgTri1 GF | AGGGAACAAAAGCTGGGTACCTGGCATTTGGCAAGTTCA | For FgTri1::GFP  construction |
| FgTri1 GR | GCCCTTGCTCACCATAAGCTTGTCATCCTGTACCAATTCCA |
| FgTri4 GF | AGGGAACAAAAGCTGGGTACCTCCCGACGATGTGGTTATAT | For FgTri4::GFP  construction |
| FgTri4 GR | GCCCTTGCTCACCATAAGCTTCAAAGCCTTGAGAACCTTGA |
| β-Tubulin F | TCTGACTTCAGGAATGGTCGTTAC | Inference primers for qRT-PCR |
| β-Tubulin R | AGCGGTCTGGATGTTGTTGG |
| *FgTRI1*qRT F | TCCAGACTACGAAGTGCTA | qRT-PCR primer for *FgTRI1* |
| *FgTRI1*qRT R | TCATCCTGTACCAATTCCAAT |
| *FgTRI4*qRT F | ACCAGGTCCTCAGTCTTG | qRT-PCR primer for *FgTRI4* |
| *FgTRI4*qRT R | TCGTTGTGCTTGCCATAG |
| *FgTRI5*qRT F | TGAGGGATGTTGGATTGAGCAGTAC | qRT-PCR primer for *FgTRI5* |
| *FgTRI5*qRT R | TGCTTCCGCTCATCAAACAGGT |
| *FgTRI6*qRT F | GCTACTCAGAATGCCCTCAG | qRT-PCR primer for *FgTRI6* |
| *FgTRI6*qRT R | CGCATGTTATCCACCCTGCTA |
